# Understanding Urban Green Space as a Health Resource: A Qualitative Comparison of Visit Motivation and Derived Effects among Park Users in Sheffield, UK

**DOI:** 10.3390/ijerph10010417

**Published:** 2013-01-22

**Authors:** Katherine N. Irvine, Sara L. Warber, Patrick Devine-Wright, Kevin J. Gaston

**Affiliations:** 1 Institute of Energy and Sustainable Development, De Montfort University, Queens Building, The Gateway, Leicester, LE1 9BH, UK; 2 University of Michigan Integrative Medicine Program, Department of Family Medicine, University of Michigan, Ann Arbor, MI 48104, USA; E-Mail: swarber@umich.edu; 3 Geography Department, College of Life and Environmental Sciences, University of Exeter, EX4 4RJ, UK; E-Mail: P.G.Devine-Wright@exeter.ac.uk; 4 Environment and Sustainability Institute, University of Exeter, Penryn, Cornwall TR10 9EZ, UK; E-Mail: k.v.frankland@exeter.ac.uk

**Keywords:** green space, health, well-being, motivation, relaxation, physical activity, environment, restoration, place, stress.

## Abstract

With increasing interest in the use of urban green space to promote human health, there is a need to understand the extent to which park users conceptualize these places as a resource for health and well-being. This study sought to examine park users’ own reasons for and benefits from green space usage and compare these with concepts and constructs in existing person-environment-health theories and models of health. Conducted in 13 public green spaces in Sheffield, UK, we undertook a qualitative content analysis of 312 park users’ responses to open-ended interview questions and identified a breadth, depth and salience of visit motivators and derived effects. Findings highlight a discrepancy between reasons for visiting and derived effects from the use of urban green space. Motivations emphasized walking, green space qualities, and children. Derived effects highlighted relaxation, positive emotions within the self and towards the place, and spiritual well-being. We generate a taxonomy of motivations and derived effects that could facilitate operationalization within empirical research and articulate a conceptual framework linking motivators to outcomes for investigating green space as a resource for human health and well-being.

## 1. Introduction

Over half of the World’s population lives in urban areas [1]. Urban living is often linked to poor respiratory health [2], sedentary lifestyle [3], increased obesity [4] and cardiovascular disease [5]. Stress and associated mental ill-health are also rising [6,7]. These trends heighten the need to identify mitigating factors and implement sustainable patterns of healthy urban living [8,9]. In seeking novel approaches to address urban health problems, there is a resurgence of interest in the role of publicly available urban nature, such as parks (also called green space), for human well-being [10,11,12]. In the United Kingdom (UK), for example, programs such as “green gym” initiatives [13] are being developed that specifically utilize green places to promote physical activity and mental health. Against an accumulating body of evidence on the benefits associated with exposure to nature [14,15,16,17] and increasing policy interest to promote the use of the natural environment to address human health/well-being [18], this paper reports on an *in situ* qualitative investigation into the extent to which health and well-being are spontaneously identified by green space users as reasons for and derived effects from the use of public green space. 

### 1.1. Background

The personal benefits of interaction with urban nature include improved cognitive functioning [19,20], reduced mental fatigue [21], increased social interactions [22,23], opportunities for reflection [24], and stress amelioration [25]. Studies linking population health with green environments demonstrate positive associations between neighbourhood green space and measures of health status [26,27], reduced odds of coronary heart disease, respiratory disease, depression and anxiety [28], and increased longevity [29]. In the UK, lower socioeconomic groups living in greener environments experience reduced all-cause mortality and mortality from circulatory disease [30]. 

Previous research into the use of parks has identified reasons for visiting that include to relax, take children outside, walk, engage in sport/exercise, experience nature, meet friends, and escape/take a break [31,32,33,34,35,36,37,38]. Identified benefits include enjoyment of being outside, social interaction, freedom, unity with nature, relaxation, and refreshment [31,33,36,37,39]. Public parks have also been identified as offering opportunities for reflection and as a source of positive emotional bonds towards and sense of identity associated with the place [24,40]. Methods of investigation have employed *in situ* observation [39,41], focus groups [37], long-term discussion groups [31], and structured questionnaires [24,33]. 

Although insight can be inferred from existing studies, the extent to which users of urban green space acknowledge or perceive personal health/well-being as a factor in their use and experience of the space is less developed. Pinder *et al.* [39], for example, identified distinct perceptual differences between managers and users of a community forest when considering the environment-health relationship. The authors highlight the need to understand how this relationship is conceptualized by ordinary green space users, noting the “relatively undeveloped theoretical models for how physical environments impact on human health, and the lack of consensus about exactly what we can and should measure” (Pinder *et al.* [39] p. 349). 

### 1.2. Existing Person-Environment-Health Frameworks

Several theories provide perspective on the person-environment-health relationship. Likewise various models are available for understanding health outcomes. These frameworks, outlined briefly below, are often studied in isolation yet, if examined together and from an interdisciplinary perspective, could potentially provide a richer explanation of observed motivations, behaviors and outcomes. 

#### 1.2.1. Attention Restoration Theory

Attention Restoration Theory (ART), a dominant model within the field of environmental psychology, proposes that the natural environment is cognitively restorative [14,42]. Underpinning ART is the hypothesis that the capacity to direct attention to one stimulus at a time (e.g., a task) requires inhibition of competing stimuli and that, over time, this capacity fatigues, resulting in mistakes, failure to focus or impatience [43,44]. Studies of brain-damaged patients support this theory, demonstrating that attentional deficits are associated with damage in the pre-frontal cortex which plays an inhibitory role in high-level mental activity like problem solving or planning [45,46,47]. Natural settings are proposed to facilitate recovery from mental fatigue through softly fascinating stimuli that are compelling without mental effort. 

#### 1.2.2. Sense of Place

Another framework for understanding the relationship between people and urban green space suggests that the experience of the place itself is psychologically important. Found across several disciplines, including geography [48], sociology [49], environmental psychology [50], and natural resource management [51], place-related theory and research focuses on attachment, *i.e.*, the emotional bonds that people have with a physical place [52,53] and identity, *i.e.*, the contribution that a place makes to one’s personal identity [54,55]. Place-related research has not typically addressed health and well-being although studies examining “favorite places” suggest that people may seek these places, particularly ordinary natural environments such as parks, for management of feelings and the self [56,57,58]. 

#### 1.2.3. Therapeutic Landscapes

Another examination of place, found in the therapeutic landscape literature emerging from cultural geography, is focused on the theoretical constructs of how places enhance health. Gesler [59] postulates the inextricability of culture, environment, and health. His examination of special healing places, such as Lourdes, France [60], identifies the interplay of the environment, the social conditions and human perception in producing the therapeutic effect. Williams [61] amplifies the importance of meaning and symbolic landscapes as she explores the contribution of therapeutic landscapes in holistic medicine. Wilson [62] highlights the concepts of identity, spirituality, and health as intimately linked to place among First Nations peoples. Further empirical research in the field has focused on the influence of social spaces such as public parks on well-being, physical activity, and stress [63,64].

#### 1.2.4. Psychophysiological Theory

The psychophysiological or stress reduction theory links nature experiences to human physiology [65,66,67]. Stress from life events or daily hassles induce a state in which the body is dominated by the sympathetic nervous system, *i.e.*, the fight or flight response [68,69]. The opposite, the relaxation response [70], mediated by the parasympathetic nervous system, explains the benefits of some health practices such as meditation. Experimental research demonstrates that being in a non-threatening natural environment reduces measures of sympathetic outflow, such as blood pressure, heart rate, skin conductance, serum cortisol, and urine adrenaline, suggesting a physical mediator of the health benefits of nature [67,71,72,73,74,75].

#### 1.2.5. Models of Health

Health has been measured by self-reported health, mortality, and specific measures of physical disease, as employed in the biomedical model. The biopsychosocial model [76,77], an expanded model including psychological health and social support or barriers, accommodates the observation that multiple factors can impact health behaviors and outcomes. With increasing recognition of a spiritual dimension, some argue for a biopsychosocial-spiritual model [78]. Additionally, research on the construct of psychological well-being identifies both a cognitive and an affective portion [79]. Thus, an expanded model of health, including five dimensions, physical (bio), cognitive, affective (psycho), social and spiritual, could become a relevant framework for assessing health effects of urban green space but has yet to be systematically applied in environment-health research.

### 1.3. Study Focus

We report here on a qualitative sub-study within an interdisciplinary, mixed methods study aimed at understanding the contribution of urban nature to human quality of life and biodiversity conservation; results of other aspects of the study have been reported elsewhere [24,41,80]. This portion of the study purposefully sought to distinguish between motivations for and derived effects from use of urban green space; to develop a weighted taxonomy representing both breadth and relative salience of comments; to examine the extent to which health/well-being is present among participant’s offered motivations and effects; and to articulate a conceptual framework that accommodates existing theory but emphasizes the words of park users in understanding the place of health in urban park usage. 

## 2. Method

### 2.1. Study Setting

Research was conducted in Sheffield, UK, population 513,000 [81]. The fifteen study sites comprised publicly accessible green spaces located along a wedge-shaped transect from the city center to the western suburbs. The sites ranged from 1–23 hectares (2–56 acres) and included parks, formal gardens, amenity green space, semi-natural areas, and school playing fields [24]. These sites also varied in ecological quality [24].

### 2.2. Data Collection and Procedure

Two open-ended questions were embedded in an otherwise structured questionnaire that explored sense of place, attention restoration, perceptions of biodiversity [24], and the soundscape [80]. The two questions were developed to capture users’ own descriptions of why they were using the space (“*And as for today, what are the main reasons that brought you to this park*”) and usual effects derived from its use (“*And thinking about after you leave this park, what words would you use to describe how you feel after you leave here*”). The visit motivation question was asked first; the derived effects question was asked second – both were asked prior to any of the closed-ended sense of place or attention restoration questions. The use of open-ended questions—in contrast to a checklist or structured scale—facilitated both capture of participants’ own words and insight into what people spontaneously identify as motivations and effects. Study materials were piloted and underwent ethics review. 

The questionnaire was delivered face-to-face *in situ* (07:00 to 18:30) on weekends and weekdays, July through October. A project description and assurance of anonymity was provided in writing and verbally prior to the interview. Consent was obtained verbally. Interviewers canvassed different locations within each green space (e.g., paths, children’s play area), approaching individuals engaged in a spectrum of activities (e.g., sitting down, walking, fishing, playing sports). In 13 sites every third individual was invited to participate; in the remaining two, due to low user numbers, all individuals were approached. Interviewers took notes on responses to the open-ended questions, obtaining participants’ own words. In the few instances in which participants self-completed the questionnaire, responses were verbatim. Where a participant gave multiple responses to a question, all were noted. Interview length for the entire questionnaire ranged from 5 to 70 min (18 min average). 

### 2.3. Participants

A total of 565 park users were invited to participate; 312 individuals took part (55% response). The sample consisted of 54% women, 87% European ethnicity, and an age range of 16 to over 70; approximately half (44%) were over 40 years old. From the 312 participants who took part, 47% were visiting alone and 36% considered themselves daily users. All 312 participants answered the visit motivation question. For the derived effects question, responses were excluded for twelve first-time users whose responses were considered speculative; seven individuals provided no response to the question. 

### 2.4. Analysis

Responses were entered into Excel spreadsheets with multiple answers to the same question treated as separate, individual statements. Two authors (KNI, SLW) conducted an iterative content analysis [82,83]. Responses were initially sorted into codes based on participants’ language; code names were developed to closely reflect the particular wording used by participants (e.g., *take a break* rather than *break*) and were based on the most frequently mentioned words/phrases. For example, the early code name of *get out/be outside* underwent revision to the final code of *get outside* because the latter phrasing was more evident across the majority of responses within this set of responses. Motivation-related codes were phrased, when possible, as verbs (to emphasize the action, e.g., *eat* instead of *meal*), highlighted the activity or attribute of the place that might bring a person to the site, and differentiated between reasons that were internal (e.g., “chill out”) as compared to external (e.g., “child wanted to play”). Derived effect-related codes were phrased as descriptive statements in an aim to differentiate effect from motivation. For example, for reasons related to fresh air, motivation responses were coded as *fresh air* while derived effect responses were coded as *full of fresh air*. These distinctions were in keeping with the way participants used language to describe their experience. 

Codes were clustered into themes. Theme labels were derived from participants’ language (e.g., most frequently mentioned word/phase within cluster of codes), findings from previous research (e.g., nature is an identified motivation for park use [33]), and theoretical constructs from existing frameworks (e.g., the label “attention restoration” [42] was used to describe the cluster of codes *better perspective*, *clear headed*, *had a break* and *motivated*). Themes were further organized into domains. Domains were informed by and grounded in constructs from place-related, attention restoration and psychophysiological theories and the biopsychosocial-spiritual health model as well as previous literature (e.g., children are an identified reason why people use urban green space [31]). 

The analysis was aided by continuous review of the participant’s own words/phrases and purposefully drew upon researchers from different disciplines (environmental psychology, health) who inherently interpreted the data differently. Participant responses were reviewed both as responses to a distinct question (*i.e.*, motivation, derived effect) as well as a combined response across the two questions, which provided richer understanding for placement. Content analysis was also aided through development of a visual mind map (FreeMind version 0.81) and the use of a thesaurus; the latter served as a method for understanding the meaning of and overlap between words for identification of nuances and distinctions in participants’ responses. This process facilitated the discussion, dialogue and consensus building that resulted in decisions on final placement of participant responses. For example, while relaxation can be considered as related to both cognitive and physical health, we decided to categorize it as physical because of the empirically identified physiological pathway by which it occurs [84]. 

Content analysis resulted in no double coding of responses which allowed quantification at all three levels—code, theme, domain—to provide a measure of salience, *i.e.*, the frequency with which a given motivation or feeling state was present among participants, and to enable comparison between the two questions. Coding resulted in 445 motivation-related statements from a raw data set of 442 responses and 527 derived effect statements from an original 532 responses. 

Within-individual comparative review between visit motivation and derived effects was conducted on a subset of responses related to the two most common codes: walking and relaxed. Content analysis further identified individual response sets highlighting the complexity of visit motivations and derived effects. For this analysis, the individual comments of users who provided multiple responses to each question were reviewed. 

## 3. Results

The interpretive analysis suggested six discrete motivational domains and seven distinct derived effect domains amongst the verbal responses provided by participants. Results are presented for each question separately followed by a comparative review. Participant comments are in quotation marks with the participant ID number in brackets.

### 3.1. Motivations for Use

The six visit motivation domains included: physical, space qualities, children, cognitive, social and unstructured time (Table 1). 

**Table 1:** Participant reasons for visiting urban green space (N = 445 comments). Park users, *in situ*, answered the question, “As for today, what are the main reasons that brought you to this park?” Codes, themes, and domains are identified along with the number of comments within each.

Domain	#	Theme	#	Code	#	Sub Code	#
Physical	232	Physical pursuits	192	Walking	163	Walk dog	65
						Passing through	45
						Route work/home	24
						A walk	20
						Pleasurable route	6
						Regular route closed	3
				Sport	19		
				Exercise	10		
		Physical restoration	40	Eat	28		
				Sit	4		
				Rest	2		
				Relax	3		
				Chill out	3		
Space qualities	111	Nature	67	Fresh air	15		
				Get outside	13		
				Sunshine	13		
				Nice weather	8		
				Fauna	9		
				Flora	6		
				View	3		
		Park features	37	Proximity	16		
				Facilities	12		
				Peace & quiet	7		
				Nice place	2		
		Place identity	4	History of use	4		
		Place attachment	3	Emotional attachment	3		
Children	49	Physical pursuits	24	Walking	2		
				Sport	5		
				Play	17		
		Social	10	With children	3		
				With adult	7		
		Nature	9	Fresh air	3		
				Get outside	6		
		Unstructured time	4	Holiday	4		
		Park features	2	Place to cycle	1		
				Open space	1		
Cognitive	25	Mental pursuits	20	Read	11		
				Purposeful work	4		
				Photography	4		
				Think	1		
		Attention restoration	5	Take a break	5		
Social	18			Meet friends	17		
				Watch people	1		
Unstructured time	10			Unstructured time	10		

The physical domain included the most common theme, physical pursuits. Walking, the most frequent of these motivations, included dog walking, passing through or going for a walk; the latter was least endorsed. Of those passing through, six individuals suggested that walking via the park was a purposeful choice as it was more pleasurable than other possible routes, e.g., “pass through to not go on main street because of cars and fumes” [P212] and “most picturesque way to walk” [P42]. All dog walk reasons were the first mentioned; for 77% of these participants (50/65) this was the only reason for coming to the park. Sport and exercise were less frequently mentioned. Within the second theme of this domain, physical restoration, the majority of comments focused on a desire for literal nourishment through eating. The remaining codes emphasized coming to the park to rejuvenate the body through sitting, resting, or relaxing. 

The space qualities domain consisted of comments pertaining to both physical and intangible aspects of the place. References to nature were noticeably present, particularly less tangible elements such as fresh air, sunshine; among the tangible, fauna (almost exclusively ducks and squirrels) were more commonly mentioned than flora. Nature was the only mentioned visit motivation for 18 individuals; thus in most instances nature was one of multiple reasons for coming to the park. For example, for a dog walker [P78] the green space also provided an opportunity to “be in the sun” while for another participant it was an opportunity for “fresh air, [to] hit a few golf balls” [P41]. Non-nature reasons for use emphasized the physical nearness of the space to one’s home or work and the available facilities, such as play areas for children, events and, for one individual, the fact that “it’s free, so might as well use it” [P78]. Comments about the peace and quietness of the place as well as “it’s a nice place to be” [P40] illustrate the more intangible components of the park features theme. Two theory-based themes, place identity and place attachment, also emerged as visit motivations capturing the identity from or feelings of attachment to the park. Mentioned minimally, these are illustrated by comments such as “[I] grew up here and played here as a child so [I am] familiar with it” [P273] and “feels like a second home” [P188], respectively. 

The third most frequently mentioned motivator was a child. Reasons for bringing children largely mirrored those that brought adults to the parks. Physical pursuits encompassed almost half the comments with “play” a recurrent activity for children. Examples include “bring children to play” [P162] or “take kids to park to let them run off energy” [P25]. For 33 individuals children were the primary visit motivation. While play dominates, social, nature, unstructured time, and park features were also present. 

The cognitive domain, fourth in endorsement, contained a theme related to purposeful mental pursuits (e.g., reading, photography); it is analogous to the physical pursuits theme that described activities that engage the body. Within this theme is a small but interesting collection of comments coded as purposeful work. For some, engagement with the green space itself constituted that purposeful work, for example, through “keeping an eye on the park, picking rubbish (e.g., broken glass) up on my route” [P277]. Only five green space users specifically mentioned coming for mental restoration, considered characteristic of Attention Restoration Theory. These types of comments were either the first or second mentioned and almost exclusively described as taking a break or getting away from work.

The fifth dimension, social, was less present as a motivator. The green space was primarily a meeting point, a place for socializing; one participant spoke of this aspect in terms of people watching, *i.e.*, “[to] look at kids playing with bikes and skates” [P2]. The social dimension was the only mentioned reason for eight participants; it was more frequently coupled with motivations from the other identified domains. For example, one participant combined social time with dog walking and bird watching: “[I come to] walk the dog, meet friends and look at wildlife, birds in particular” [P247].

The final domain to emerge from participants’ comments was unstructured time. While it had the fewest number of comments, the motivation described was distinct. One participant described this as “wander through woods...today I decided to just explore and see where paths lead me” [P203]. The comments within this domain suggested a purposefully unplanned period of time.

### 3.2. Derived Effects

Participants’ 527 comments describing how they feel after leaving the park were grouped into seven domains: physical, affective, place attachment, spiritual, cognitive, global well-being, and social (Table 2). 

Most participants identified benefits in the physical domain, which included themes associated with the physical body. “Relaxed” was the single most commonly mentioned word although some participants provided greater imagery, such as “wound down a bit” [P82] and “bit mellow because of greenery in the city” [P30]. Over three-quarters (85%) of comments within the relaxed theme were mentioned first. The revitalized theme, made up of four codes, was clearly distinct in tone and is captured by comments such as “recharge your battery” [P111] or “[a] bit more energized; revived having had fresh air/exercise” [P47]. While most individuals felt positive physical states, a few felt depleted (e.g., “physically exhausted as I always run around when here” [P275]) or uncomfortable.

**Table 2:** Participant specified derived effects following green space use (N = 527 comments). Park users, *in situ*, answered the question, “Thinking about after you leave this park, what words would you use to describe how you feel after you leave here?” Codes, themes, and domains are identified along with the number of comments within each. Comments included in the analysis were from individuals who had visited the given green space previously.

Domain	#	Theme	#	Code	#
Physical	217	Relaxed	126	Relaxed	118
				Chilled out	8
		Revitalized	52	Refreshed	30
				Energized	10
				Full of fresh air	8
				Exercised	4
		Depleted	28	Tired-physical	17
				Hungry/thirsty	9
				Tired-vigilance	2
		Comforted	9	Rested	6
				Warm	2
				Well fed	1
		Uncomfortable	2	Uncomfortable	2
Affective	102	Positive emotions	59	Happy	26
				Good/fine/nice	21
				Pleasant	12
		Neutral feelings	22	Normal	13
				No change	9
		Intensely positive emotions	13	Wonderful	5
				Exhilarated	4
				Joyful	4
		Don’t know how I feel	8	Don’t know how I feel	8
Place attachment	74	Value of park	43	Contrast from city	12
				Nice experience	10
				Sad because leaving	7
				Nice space	5
				Enjoyable place	4
				Upset about park condition	5
		Appreciation	24	Appreciation	7
				Lucky	4
				Glad	4
				Pleased	3
				Grateful	3
				Love	3
		Relieved because leaving	7	Relieved because leaving	7
Spiritual	68	Tranquil	58	Calm	23
				Peaceful	20
				At ease	6
				Tranquil	4
				Serene	3
				Quiet	2
		Interconnected	10	Been outside	6
				Connected to nature	4
Cognitive	44	Satisfied	26	Job done	15
				Satisfied/content	11
		Attention restoration	17	Had a break	6
				Clear headed	4
				Better perspective	4
				Motivated	3
		Rushed	1	Rushed	1
Global well-being	16			Better	9
				Healthy	7
Social	6			Connected to others	6

Two domains, affective and place attachment, together represented park users’ emotions, either within oneself or feelings about the park. The majority of personal feelings were positive (e.g., happy, good) although some people felt strongly positive, such as joyful or exhilarated. Exemplar comments from the affective domain included, “come up feeling fed up, leaving feeling great” [P241] and “happy because [you] have seen wildlife [you] would not have seen at home” [P187]. Comments capturing the emotions toward the place included, “I just love it, even on cold, rainy days” [P61] and “[I] considered joining a gym but realized it was more enjoyable to come to the park for walking” [P187]. For some participants the resultant emotional state was contingent on context, such as another’s behavior, the activity, or the weather. A minority of individuals had neutral (e.g., “not life changing, it’s convenient” [P32]) or, more rarely, negative feelings. The latter were generally based on either the deteriorated condition of the park, such as the participant who was “disappointed, upset by condition of park” [P34] or a perceived lack of safety as noted by an individual who felt “relieved because it can be unsafe and isn’t welcoming” [P272]. 

A spiritual domain emerged as the fourth most commonly mentioned derived effect with comments organized into two themes. Expressions of tranquility, made up of six codes, reflected a sense of peace akin to being in a sacred place; examples of more detailed responses included “calms you down—out of hustle and bustle” [P92] and “go from peace to rat race; feel peaceful here” [P94]. The experience of being unified with and a part of a larger reality was found in comments about a connection to nature (e.g., “[I] am left with images of green” [P138]) and the euphoric feeling of being outside of the built environment (e.g., “the feeling of just getting outdoors!” [P109]) is an exemplar. 

Use of urban green spaces contributed to cognitive health through providing opportunities for satisfaction and mental restoration. Satisfaction related to accomplishing a specific task (e.g., walk the dog) or a sense of contentment with how one has spent one’s time (e.g., “[I came because] off work, lovely weather, [to] read a book [and left] satisfied with what I did with day” [P117]). Fewer comments directly pertained to the restoration of attention. This effect was captured by the comment, “got things in perspective because I’ve done a bit of mulling things over and everything is put in its place after being here” [P217]. 

A small number of participants conveyed a sense of global well-being. The unspecified nature and broad content of the comments associated with two codes within this domain (better, healthy) suggested that an important general effect was occurring. For example, one participant stated they “feel a bit better even if quite [a] subtle feeling” [P276] while another left “look[ing] forward to next time I will come to park, [I] feel healthy, [I’ve] had a walk, [and I’m] happy” [P40]. A further examination of instances where participants gave several answers showed that many were describing multiple aspects of health. For example, one individual felt physical effects of “more relaxed, refreshed really” and mental stimulation, *i.e.*, “find it quite interesting—always notice something new” [P208]. Another person experienced spiritual, physical, and emotional well-being: “peace of mind, relaxing; listening to birds, watching wind, birds fly kind of calms me; feel fantastic, had a bit of fresh air” [P113]. Perhaps the clearest statement that encapsulated this holistic health effect was from the individual who came to go “jogging” and whose derived effect description touched on almost all health domains: “relaxed, quite joyful, peaceful, despite noise one hears in park, quite clear-headed actually, therapeutic—being here is therapeutic” [P20]. 

Only six participants mentioned the social benefit of feeling connected to others. One individual, who came for the facilities, expressed this effect as “caring—feel more inclined to think about people as individuals rather than just lump together in a category, particularly in terms of teenage boys in skatepark” [P66]. 

### 3.3. Comparative Review

**Figure 1:**
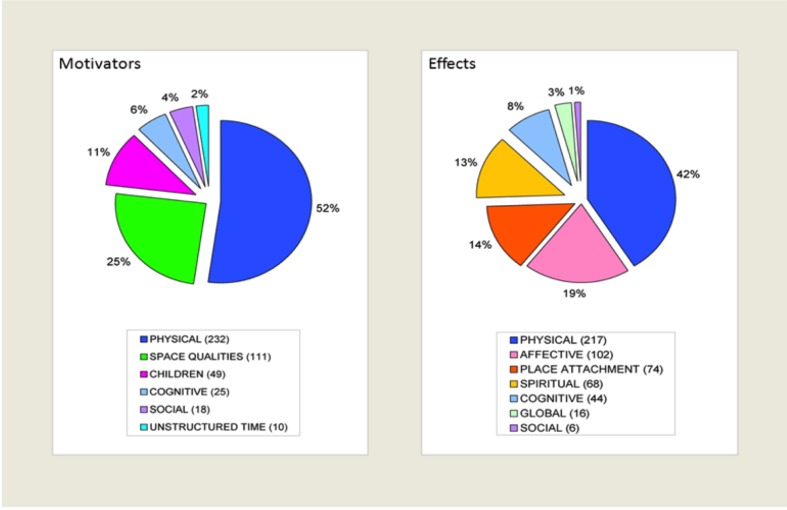
Relative endorsement of visit motivation for and derived effects from the use of urban green space. Domains associated with visit motivation (**left**) and derived effects (**right**) are depicted along with the number and percent of comments associated with each domain.

A comparison between the identified domains (Figure 1) provided insight into the breadth and salience of reasons why urban green space was used and the effects derived from that use. 

The physical body domain was the largest for both, representing 52% of visit motivation (mainly walking) and 41% of derived effects (mainly relaxed) responses. Cognitive and social domains were also jointly present. In both instances these were a small proportion of responses. Attention restoration was an identified visit motivation for five individuals while only 17 indicated it as a derived effect. Place attachment, a fairly insignificant visit motivation theme, became a notable post-park effect domain comprising 14% of the overall responses. Children, a prominent visit motivation, appeared only as explanations for accomplishment or satisfaction, captured by codes within the cognitive derived effect domain. Nature, a central visit motivation theme within the space quality domain, was located at the derived effect code level within the themes of revitalized (*full of fresh air*), interconnected (*connected to nature*), and value of park (*nice space*). The affective and spiritualdomains (19% and 13% respectively) emerged only as effects derived from park use; affective states were largely positive (70%). Additional differences between visit motivations and effects included the presence of unstructured time as a driver (2%) and global well-being (3%) as a derived effect. 

To further understand users’ experiences, we examined the most frequently mentioned visit motivation, walking, and the most common effect, relaxed, using a within-subject review. Walking was identified as a major driver for green space use and, for most (144/163, 88%; Table 1), it was their primary reason. An examination of the relationship between visit motivation and the derived effects identified that the foremost state after walking was positive, including relaxed, having positive emotions about themselves and the park, feeling tranquil, revitalized, and satisfied. For example, among the dog walkers, one individual described their feelings after leaving the park as “serene, peaceful; as if [I’ve] brushed the cobwebs off; had lots of fresh air; [P74]. Another, going home through the park, felt “more peaceful, energized, more in touch with natural world, glad it is here” [P290]. For an individual who specifically came for a walk, they stated “I feel pleasant, feel a rosy glow like you’ve read a good book, for a while you are missing the exhaust [and] the noise” [P120]. These quotes suggest a link between the physical pursuit of walking with resultant positive and multi-dimensional states of well-being. 

Relaxation was most strongly present in how park users felt afterwards. For 79% (100/126; Table 2) relaxed was the primary feeling experienced yet only two of these individuals specifically mentioned relaxation as their primary motivation for coming to the park. The vast majority came to the park for other reasons. For example, an individual who came for “good weather” indicated feeling “relaxed, wonderful; sad that I have to go back to work” [P69] when they left the park while another who came for “dog walk, children out to play, get some fresh air” left the park feeling “relaxed, exhilarated” [P298]. Several participants’ feeling of relaxation was also explicitly linked to statements of holistic health. For example, one came for “…some fresh air, read a book, sunshine, peaceful, free exercise” and then left “relaxed, feel a lot healthier, got a smile on my face, serene” [P205]. 

## 4. Discussion

The focus of this study was to understand the place of health and well-being in urban park usage. Comments from urban green space users *in situ* illustrate the breadth, depth and salience of the reasons for park use and the effects experienced. Statements about visit motivations were divided into six domains ordered by frequency of endorsement: physical, space qualities, children, cognitive, social, and unstructured time. Prominent motivators in each domain included walking, intangible elements of nature (fresh air), play for children, purposeful mental pursuits, social gathering, and purposefully unplanned time. Seven domains described the derived effects: physical, affective, place attachment, spiritual, cognitive, global well-being, and social. Participants identified feeling physical effects such as relaxation and revitalization, positive emotions, attachment towards the place, spiritual tranquility and connection, cognitive satisfaction, an overall sense of health, and social connection. An examination of the relationship between visit motivation and the ensuing state identified that walking resulted in largely positive effects across multiple domains including physical, affective, place attachment, spiritual and cognitive. Relaxation, while rarely cited as a visit motivation, was central to how park users felt afterwards and was associated with a wide range of visit motivations. Our analysis identified the complexity of motivations for park use and the multiple aspects of individual well-being that can result from time in urban green space. 

### 4.1. Visit Motivation

Visit motivations provided by participants in this study broadly mirror findings from previous research into the use of urban green space worldwide: UK: [31], Netherlands: [68], Turkey: [33,38], China: [35], Iran: [36]. Walking, exercise, taking children to play, getting outside, fresh air, observing nature, having a break, meeting friends, and unstructured time are all reasons that thread through these studies as motivations for park use. The methodological differences across studies, however, make it difficult to gauge the weight, breadth and depth of individual reasons. Divergence is present in the focus of questions posed, the wording of reasons provided in checklists and data collection methods. For example, focus group-based methods identified social interaction as a central driver for park use [37] yet, when included in a closed-ended checklist, this same item is infrequently selected [33]. A low frequency of mention was found in this study as well. We also note that in studies using checklists, 50% or more of participants has identified relaxation as a motivator [33,35,36] whereas in this study relaxation was rarely mentioned as a reason for visiting but emerged strongly as a derived effect. 

The qualities of the place itself, the majority of which were nature-related comments, were clearly important reasons for park use among participants in this study. While in keeping with other studies [33,35], by staying close to participants’ own words we identified an important breadth to the “experience of nature”; fresh air, getting outside and sunshine emerged more strongly than flora and fauna suggesting that these intangibles are highly valued. Proximity and specific facilities were also important drivers for use, providing support for the emerging literature on the relationship between park characteristics and physical activity [85,86]. 

Concepts from sense of place literature and Attention Restoration Theory featured minimally as participant identified motivators. The few comments within the cognitive dimension were largely represented by pursuits that involve the mind (reading) with only five individuals specifically identifying the desire for a break. This differs from studies of urban parks that have used closed-ended checklists in which the opportunity to “escape the city” [33] or “get away from stressful environments” [36] were identified by a third of the participants; focus groups have also identified “getting away from it all” as a common theme [37]. Clearly some comments within the physical restoration visit motivation theme (related to resting, sitting, relaxing, and chilling out) might be construed as taking a break; we have however interpreted those as related to the physical body rather than the mind, based on the descriptive physical nature of participants’ wording. 

The interesting presence of a desire for unstructured time, for both adults and children, perhaps highlights recognition of its rarity in our modern world [87,88]. Its identification as a visit motivation for adults mirrors research on the role of accessible, nearby nature for teenagers as a place to hang out alone or with friends, explore and develop a sense of identity [89,90]. 

As a whole, visit motivations minimally identified the use of urban green space for the explicit “pursuit of health”. Participants never used the word “health”, and while physical pursuits dominated the coded responses, most of which centered on walking, walking in parks was more often incorporated as part of a daily routine rather than a purposeful health walk. Our findings remind us of the multiplicity of ways in which ordinary park users conceptualized park use, highlight the minimal explanation provided by any one theory, and underscore the need for a fuller consideration when measuring—and seeking to promote—urban green space for healthy living.

### 4.2. Derived Effects

Comments associated with having been in an urban green space described a complex set of effects usually experienced by the park users in this study. The major derived effect was “relaxed”, a physiologic state in which the person experiences decreased respiratory rate, heart rate, and muscle tension [84], which is associated with enhanced mood and improved work performance [91]. Relaxation buffers stress and emotional problems, improves immune system function, and alters genetic expression [92,93,94] and can be produced by various health practices (hypnosis, meditation) as well as by the tradition of nature mystics, who immerse themselves in the quiet of nature [84]. In this study, individuals experienced relaxation by visiting an urban green space for a multiplicity of reasons, which suggests a potential physiological mechanism through which nature has its effects on our physical well-being [66,67]. The corollary, *i.e.*, stress levels, has been investigated by other researchers such as a large-scale Danish study [95] that found an association between distance to publicly accessible green space and experienced stress levels. 

The identified broad spectrum of positive emotions relates to empirical work in the emerging field of positive psychology [96] which has linked positive emotions with resilience [97], human psycho-social flourishing [98] and longevity [99,100]. As benefits of positive emotions become better understood, interventions to produce them will likely become of interest; our findings suggest that visiting urban green space allows people voluntarily to increase their experiences of positive emotions and potentially identifies another important mechanism underlying the effects of green space on well-being. 

The other affective component identified in this study is place attachment including positive feelings towards the green space, such as love or gratitude. The experience of love or gratitude, themselves positive emotions, has been found to promote individual well-being [101,102,103] thus potentially amplifying the beneficial effects of positive emotions experienced in green spaces. It is also consistent with Tuan’s concept of topophilia [48] and the sense of place literature [50] with specific parallels to the work of Korpela and Ylen [58] on the relationship between natural favorite places and self-regulation of mood. Our findings also provide further insight into how the place attachment construct is described by ordinary park users which may facilitate development of standardized measures [24,40].

The expressed feelings of tranquility and interconnectedness, interpreted here as spiritual and previously documented to occur in wilderness settings [104], suggests that even urban green space can enhance these aspects of spiritual well-being. This is consistent with Wilson’s [62] explication of the importance of spirituality in relationship to the landscape among First Nations’ peoples. In our study, seeking spiritual well-being was not a visit motivator, as has been identified in other studies using closed-ended questions [105], but rather an unanticipated benefit of use. 

Comments pertaining to cognitive health emphasized the satisfaction gained from having achieved one’s aims and from how one has spent time, implying a “fit” or “match” between what the setting offers and one’s purposes. This is illustrative of the concept of compatibility, posited as an important component of a cognitively restorative environment [14,42]. However, few participants specifically identified effects that suggest mental restoration occurred—a sense of having had a break, of clearing one’s head and gaining perspective. The small proportion of participants spontaneously volunteering restoration as either a motivator or a benefit is in contrast to other studies that, when using closed-ended questions, often identify attention restoration as important [106]. Also in contrast to previous studies on urban green space [31,64] and some therapeutic landscape literature [63,64], social connectedness and support were infrequently offered spontaneously as a derived effect. 

In general, our data support a derived effect of positive well-being for individuals. Wellness has been defined as “a condition obtained when a person achieves a level of health that minimizes the chances of becoming ill…achieved by a combination of emotional, environmental, mental, physical, social, and spiritual health” [107]. This definition expands the biospychosocial model [76] and parallels the domains used in interpreting our data. The parks provide positive emotions, a positive environment filled with fresh air, mental satisfaction and restoration, physical relaxation and revitalization, social connection, and elements of spiritual health. Being in a park improves global wellness as acknowledged by participants in single comments about being “healthier” or in groups of comments that tap several health domains. Previous epidemiologic research found that walkable green spaces enhance longevity [29] and that the proportion of nearby green space is associated with improved general health [27,108]. Maas *et al.* [109] found that physical activity did not account for the relationship between green space and health, leaving open the question of what variables determine the relationship. Our research suggests that when seeking to identify and quantify health benefits of urban nature, we must purposefully measure the multiple aspects of individuals’ holistic well-being in order to understand the full impact of this simple decision: to go to the park. 

### 4.3. Integrated Conceptual Framework

**Figure 2:**
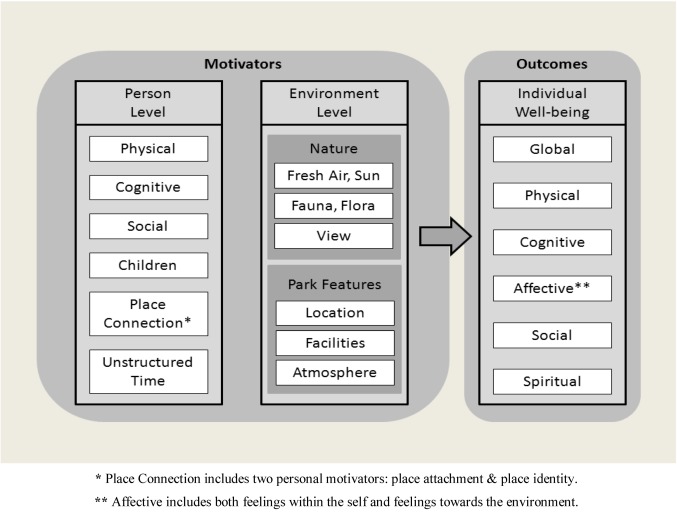
Measurement Model for Person-Environment-Health Relationship. The model distinguishes between the motivations for use of and the potential derived effects from interaction with the natural environment, providing a framework for measurement of health/well-being.

Our findings suggest the need to use a broad conceptual framework for considering the relationship between personal motivators, the natural environment, and individual health, one that takes into account the reports of green space users and is reflective of holistic benefits in multiple dimensions for the individual: physical, mental, emotional, social, and spiritual (Figure 2). 

We emphasize here that when users of urban green space are directly asked to identify their motivations or how they feel after being in the space, they give more reasons and benefits than suggested by any one existing theory or identified through the use of a closed-ended checklist drawn from previous research. Our findings suggest that theories and models may need to be combined to understand the range of motivations and effects for the individual. Understanding these components can inform future research by suggesting the selection of existing validated scales or the need for newly constructed scales to test hypotheses derived from the model. The results of this study also suggest a conscious parsing of motivators and effects that have been confounded in previous studies using structured scales. This is indeed one of the strengths of qualitative research that calls us to generate new theory or models to more fully explain the phenomena under study. 

### 4.4. Limitations

Limitations stem from decisions related to sampling, the data recording method, interpretation of the data, and the research setting. Ours was a purposive sampling of actual green space users. The sample size and the diversity of responses did not allow investigation of socio-demographic (e.g., gender) or use (e.g., visit frequency) differences. Data, collected primarily as written notes by the interviewer, may have lost some richness of the participants’ language. Inter-rater reliability was not calculated to assess our coding scheme and some comments could be interpreted differently (e.g., calm could be a cognitive element, we interpreted it as spiritual). The data were, however, examined using an iterative process that sought to resolve differences in the conceptual mapping of quotes between researchers from different disciplines (environmental psychology, medicine). Lastly, findings clearly pertain to the individuals using the park settings being studied and the limited time frame of “today” thus the research does not address historical or socio-cultural use of public spaces.

### 4.5. Future Research

Important directions for future research include a larger sample to allow analyses of group differences and further examination of the dynamic relationship between visit motivation and the post-park experience. Different cultural contexts and different types of natural environments would provide insight into the universality of the findings. Research could also usefully focus on disentangling distinctions between concepts such as a general love of nature and a love of a specific place. A crucial way to advance the field is measure development, including standardized closed-ended self-report questions that reliably measure the range of identified health domains as well as *in situ* measures of relaxation (both self-report and objective). 

### 4.6. Implications

Our results fit into a wider picture of efforts to promote healthy behaviors and plan sustainable urban environments. Although we recognize that further investigation to establish linkages would be necessary, our findings offer some suggestions worthy of inclusion in the dialogue on these matters. 

#### 4.6.1. Health Promotion Efforts

Physical pursuits, particularly walking, were the most often stated reason for visiting a park. As a low cost antidote for increasingly sedentary urban lifestyles, park-based physical activity could contribute to addressing the obesity and overweight epidemic for both adults and children [110] as well as risk of cardiovascular disease. Urban green space was not only a place for walking but also identified as a place that provided relaxation. Recent experimental work postulates a neurobiological link between stress, anxiety and depression [111] suggesting that contact with urban green space could play a role in addressing issues of mental ill-health. Our findings of improved positive emotions and spiritual well-being may also have relevance in combating depression and anxiety [97,112,113] and suggest a mechanistic path to account for the observation of decreased depression and anxiety when living near more green space [30]. Clinically, more healthcare providers could consider writing “park” prescriptions [114], emphasizing the variety of reasons for park use and the multiplicity of potential holistic health effects. Our participants also identified qualities of places that leave people feeling healthy that could be incorporated more widely into the design of health care settings, e.g., fresh air, feeling the sunshine, peace and quiet, a beautiful view along with paths that bring people through and to nature. 

#### 4.6.2. Urban Design

Urban green space is emphasized in policy documents across the world and is considered a key dimension of the sustainable city [115,116,117]. This study suggests qualities and configurations of urban green space that might promote use as well as health. For example, proximity was important for use among our participants; recent studies linking proximity of green space to health status [27,30,108,118] and a potential correlate of physical activity [85] support preservation and creation of locally integrated green places. Similarly, the centrality of walking as a motivator highlights the importance of paths through, to and between urban green spaces. Renewed emphasis on facilities for children, places to eat, sit and exercise, should be balanced with the intangibles of peace and quiet, fresh air, and tranquillity. The presence of negative feelings associated with upkeep and safety implies park management issues. Finally, identification of unstructured time as a visit motivation warns against overemphasizing physical activity as the focal point for park visitation; there is value in “just being”. 

## 5. Conclusions

Much has been written about the importance of incorporating nature into the built environment from the perspective of cultural and societal values [119,120], economic valuation [121,122], ecosystem function [123,124], ease of access across the landscape [125,126], and the experience itself [14,24,41,80]. Results from this study, derived from the comments of urban green space users *in situ*, provide a weighted taxonomy that illustrates the breadth, depth and salience of the reasons people use parks and the effects experienced. People choose to visit urban green space for many reasons, including both their own personal motivators and ones related to the space itself; healthful physical activity is but one of these motivators. Green space has the potential to provide several types of holistic health benefits including relaxation, positive emotions about self and environment, tranquility, revitalization, and satisfaction. The dominant benefit identified is relaxation and, given its demonstrated importance in health promotion, more research is warranted to understand this process. Efforts to investigate or promote urban green space as a health resource need to carefully account for the multiplicity of motivations and benefits identified by users. The preservation and expansion of green space could be framed as a health benefit to individuals, which may provide an additive rationale for environmentally sound policy decisions. 
